# Summit on cell therapy for cancer: The importance of the interaction of multiple disciplines to advance clinical therapy

**DOI:** 10.1186/1479-5876-9-107

**Published:** 2011-07-08

**Authors:** Cornelis JM Melief, John J O'Shea, David F Stroncek

**Affiliations:** 1 Department of Immunohematology and Blood Transfusion Leiden University Medical Center 2300 RC Leiden, the Netherlands; 2 Molecular Immunology and Inflammation Branch National Institute of Arthritis and Musculoskeletal and Skin Diseases (NIAMS) National Institutes of Health (NIH) Building 10, Room 6N204 Bethesda, Maryland 20892, USA; 3 Department of Transfusion Medicine Clinical Center, NIH 10 Center Drive-MSC-1288 Building 10, Room 3C720 Bethesda, Maryland 20892, USA

## Abstract

The field of cellular therapy of cancer is moving quickly and the issues involved with its advancement are complex and wide ranging. The growing clinical applications and success of adoptive cellular therapy of cancer has been due to the rapid evolution of immunology, cancer biology, gene therapy and stem cell biology and the translation of advances in these fields from the research laboratory to the clinic. The continued development of this field is dependent on the exchange of ideas across these diverse disciplines, the testing of new ideas in the research laboratory and in animal models, the development of new cellular therapies and GMP methods to produce these therapies, and the testing of new adoptive cell therapies in clinical trials. The Summit on Cell Therapy for Cancer to held on November 1 and 2, 2011 at the National Institutes of Health (NIH) campus will include a mix of perspectives, concepts and ideas related to adoptive cellular therapy that are not normally presented together at any single meeting. This novel assembly will generate new ideas and new collaborations and possibly increase the rate of advancement of this field.

## Review

On November 1 and 2, 2011 at the National Institutes of Health (NIH) campus in Bethesda, Maryland a multidisciplinary summit of laboratory and clinical investigators and individuals involved in the clinical use, manufacture, evaluation and regulation of cellular therapies for the treatment of cancer will meet to discuss the most recent advances and promising cellular therapies of cancer (http://www.sitcancer.org/meetings/am11/summit11). The meeting is sponsored by the Society for Immunotherapy of Cancer (SITC). The purpose of this Summit is to bring clinical and laboratory investigators and those involved with producing, assessing and regulating cellular therapies, together to present and discuss important scientific and technical advances that currently or will soon impact the field.

The Summit is important because this field is moving quickly and the issues involved with its advancement are complex and wide ranging. Regular, more focused immune therapy of cancer meetings remain important and, in fact, are critical to the advancement of adoptive cellular therapy of cancer, but this and most other areas of clinical therapy will benefit from the cross-fertilization that results from the interactions with other related clinical fields, regulatory agencies and industry.

While immunology, cell biology and cancer biology have been the cornerstones of adoptive cellular therapy, gene transfer, cell reprogramming and stem cell biology are emerging as important contributors to this field. All of these areas will be discussed at the Summit on Cell Therapy for Cancer. The meeting will include lectures on adoptive cellular therapy using tumor infiltrating lymphocytes (TIL), cytotoxic T cells and natural killer (NK) cells, reprogramming immune and stem cells, new methods for cell expansion, regulatory considerations and bringing new technologies from the research laboratory to the clinic.

The clinical promise of cellular therapies is growing rapidly. The treatment of metastatic melanoma with TIL, which was pioneered by the Surgery Branch, NCI, NIH, is becoming more effective and its use is becoming more widespread. Since TIL were first used to successfully treat melanoma is 1988 [1], several improvements have been made. Preconditioning patients with lymphocyte depleting chemotherapy increased the proportion of patients with objective clinical responses to 50% [2]. Further intensification of the lymphocyte depleting preconditioning using cyclophosphamide, fludarabine and total body irradiation (TBI) along with marrow rescue by the administration of autologous CD34+ isolated from G-CSF mobilized peripheral blood stem cell products improved objective clinical response rates to 72% [3,4]. Several institutions are now using TIL to treat melanoma [4-7]. Other groups have used expanded antigen specific CD8+ T cells for adoptive cellular therapy of melanoma [8-11]. Some investigators are using autologous dendritic cells or artificial antigen presenting cells pulsed with tumor antigens to expand cytotoxic T cells for melanoma therapy [8,9].

Immune therapy of cancer has spread well beyond the treatment of melanoma. The field of hematopoietic stem cell transplantation (HSCT) is moving from a cell replacement therapy to an adoptive cellular therapy. In fact, in many respects the fields of immune therapy of cancer and HSCT are merging. The non-myleoablative chemotherapy and TBI regimen and autologous CD34+ cell rescue used as part of adoptive cellular therapy protocols used to treat metastatic melanoma are similar to those used for HSCT. For many years lymphocytes collected from HSCT donors have been infused following HSCT as an adoptive cellular therapy to treat leukemia relapse following transplantation; particularly chronic myelogenous leukemia [12]. Lymphocytes from the HSCT donor are also being used to treat Epstein-Barr virus (EBV) associated B cell lymphoproliferative disease in HSCT recipients. These post-transplant lymphoproliferative diseases (PTLDs) occur most often in recipients of T cell depleted grafts. PTLD can be treated with the infusion of unmanipulated donor lymphocytes, but this is associated with a high risk of graft-versus-host disease (GVHD). In order to avoid GVHD, PTLDs are being treated with donor derived EBV-specific T cells [13,14]. These EBV-specific T cells are generated by culturing donor peripheral blood mononuclear cells (PBMCs) with EBV-transformed lymphoblastoid B cell lines (LCL) which effectively express EBV antigens and function as antigen presenting cells. Treatment of PTDL with EBV-specific cytotoxic T lymphocytes (CTLs) is effective in more than 80% of patients, and when used prophylaxtically in high risk patients is effective at preventing PTDL [15].

A number of groups are investigating the use of T cells specific to the leukemia antigens such as Wilms tumor 1 (WT1) [16,17] and proteinase 3 (PR3) [18] to prevent or treat leukemia relapse following HSCT. In addition, recently, vaccination has been able to induce robust T cell responses against cancer-associated antigens such as viral oncogenic proteins [19]. This offers the prospect to combine proper vaccine strategies with adoptive transfer of specific T cells to achieve optimal T cell expansion and therapeutic benefit [20].

Adoptive cellular therapy protocols have also begun to use NK cells. Clinical investigators interested in treating both cancer and hematologic malignancies and leukemia have been using both allogeneic and autologous natural killer (NK) cells as adoptive cellular therapy. To treat disease relapse in HSCT recipients with hematologic malignancies NK cells from the HSCT donors are being administering post-transplant. Peripheral blood mononuclear cells (PBMCs) collected from the HLA-matched donors are enriched for NK cells by the depletion of CD3+ T cells using anti-CD3 immunomagentic beads or by CD3+ T cell depletion followed by CD56+ cell selection [21]. The allogeneic NK cells are administered to the recipient at the time of disease relapse. The NK cell recipient is immunosuppressed and treated with IL-2 to allow for *in vivo *NK cell expansion. This NK cell therapy has resulted in complete hematological remission in 5 of 19 patients treated with acute myelogenous leukemia [22]. Similar NK cell preparations and treatment protocols have been used to treat patients with recurrent breast cancer and ovarian cancer [23] and refractory lymphoma [24]. The patients were given a lymphodepleting preparative regimen and were then treated with NK cells from HLA haplotype identical donors followed by 6 doses of IL-2 therapy. Among the 6 patients with refractory lymphoma, 4 have had objective clinical responses [24].

Other investigators are using *ex vivo *expanded autologous NK cells as primary therapy for cancer [25]. Autologous NK cells were isolated using a two step process from PBMC products collected from the patient by apheresis. PBMCs in the apheresis product are depleted of T cell using anti-CD3 immunomagentic beads and then NK cells are selected using anti-CD56 immunomagentic beads. The isolated NK cells are then expanded by incubation with lymphoblastoid cell lines (LCLs) as feeder cells and IL-2. The autologous expanded NK cells are being used to treat patients with advanced malignancies [26].

Gene therapy is becoming an important part of cellular therapy for cancer and hematologic malignancies, particularly, dendritic cell (DC) therapy. DCs have been used in many clinical trials of immunotherapy for cancer. For these studies DCs are usually generated by incubating peripheral blood monocytes with the differentiating agents IL-4 and GM-CSF to produce immature DCs (iDCs) which are used for some clinical trials, but for most trials iDCs are incubated with maturation agents to produce mature DCs (mDCs) [27]. Typically, DCs are loaded with immune dominant peptides or proteins prior to their administration [8,9], however, many clinical trials are now using genetically engineered DCs to epitope-load HLA antigens [17]. For many years adoptive cellular therapy using genetically engineered cells has been used to treat Hodgkin's lymphoma. The same EBV-specific CTLs that have used to treat PTLD have also been used to treat EBV-positive Hodgkin's Disease (EBV-HD) [14]. While some patients responded to this therapy, the frequency of T cell clones recognizing the EBV antigen expressed in Hodgkin's disease, LMP2, is low. As a result LMP2 specific CTLs were generated by first culturing T cells with DCs transduced with recombinant adenovirus encoding LMP2A followed by expansion by culture with LCL transduced with the same vector [14]. The treatment of 6 patients with high risk EBV-associated lymphomas in relapse with these LMP2 specific CTLs resulted in clinical responses in 5 patients [28].

Another very promising use of genetically engineered cells for the treatment of cancer involves arming autologous T cells with T cell receptors (TCR) that have a high affinity to cancer antigens, expanding these genetically modified T cells *in vitro *and infusing them into patients [29]. T cells transduced with a high affinity TCRs for the melanoma antigens MART-1 and gp100 are being used to treat patients with metastatic melanoma [30,31]. Clinical responses were seen in 19% and 30% of patients [30,31]. In addition, high affinity TCRs specific for NY-ESO-1, a cancer antigen expressed by approximately 80% of patients with synovial cell sarcoma and 25% with melanoma, are being transduced into autologous T cells, the T cells are being expanded *ex vivo *and used to treat patients with metastatic synovial cell sarcoma and metastatic melanoma [32]. This therapy has resulted in objective clinical responses in 4 of 6 patients with synovial cell sarcoma and five of 11 patients with melanoma [32].

Chimeric antigen T cell receptors (CAR) are also being used in adoptive cell therapy of cancer. One CAR that has been tested clinically is made up of the antigen recognition portion of CD19, the zeta chain of the T cell receptor and a portion of the co-stimulatory molecule CD28. Autologous T cells transduced with anti-CD19 CAR are cytolytic to B cell lymphoma cells that express CD19 [33]. While clinical trials of these genetically engineered T cells are just beginning, preliminary results have been encouraging [34].

In order to improve the effectiveness of adoptive cellular therapies with engineered T cells clinical investigators have turned to stem cell biology. While any population of CD8+ T cells can be genetically engineered; naïve, central memory or effector memory cells, engineered T cells produced from these three different types of T cells may not be equally effective in treating cancer. Restifo and colleagues have recently shown that naïve T cells are more capable then central or effector memory T cells of expressing TCR transgenes and *in vitro *expansion. Furthermore, expanded naïve cells express lower levels of markers of effector differentiation which has been associated with greater adoptive cellular therapy effectiveness and higher levels of CD27 and longer telomeres, which suggests that these cells have a greater *in vivo *proliferation potential [35]. The number of naïve T cells in the circulation varies among healthy subjects [35] and the levels of circulating native T cells are likely to be more variable among cancer patients due to prior cancer chemotherapy or the cancer itself. Patients who have had extensive chemotherapy may have very low levels of circulating T cells. As a result, investigators are working on cell reprogramming strategies to produce naïve and stem T cells for adoptive cellular therapy.

Patients are not served until new therapies are brought to the clinic. Fortunately, the clinical success of adoptive cellular therapies currently in clinical trials is driving the development of new cell production technologies that will make adoptive cellular therapy more feasible. Investigators at Baylor have found that gas-permeable flasks (e.g., G-Rex flasks, Wilson Wolf Manufacturing, New Brighton, MN) can be used to expand cytotoxic T cells to a much higher concentration than bags or traditional flasks [36]. Cell culture in G-Rex gas-permeable flasks requires approximately one-fifth the quantity of media, AB Serum, IL-2 and anti-CD3, and less equipment than culturing in bags or flasks. This reduction in culture volume and media is extremely important to producing the 10 to 40 × 10^9 ^cell used for adoptive cytotoxic T cell and NK cell therapy. Several groups are currently working to develop and validate methods to expand, TIL, T cells, engineered T cells and NK cell in G-Rex gas-permeable flasks. If the promising preliminary results of T and NK cell growth and expansion in G-Rex flasks continues, these methods will likely lead to the more widespread use many adoptive cellular therapies. Cell therapy laboratories are also working with the manufacturer of the G-Rex flasks, Wilson Wolf Manufacturing, to produce a larger gas-permeable flasks specifically designed for good manufacturing practice (GMP) cell growth that will further simply TIL, T cell and NK cell production.

Adoptive cell therapy requires cell growth and culture in multiple types of flasks and bags, with a variety of growth factors, cytokines and antibodies. Cell selection or depletion using monoclonal antibodies or elutriation is often used. Cells are sometimes transduced with retroviral or lentiviral vectors. Bringing these complex therapies to the clinic requires investigators to address a number of issues related to the safety and effectiveness of the final product. The United States Food and Drug Administration (FDA) is a critical partner in bringing safe and effective adoptive cellular therapies to the clinic and addressing the complex regulator issues related to cellular therapies. Representatives from the FDA should be included in forums that address important issues in the field of adoptive cellular therapy of cancer.

The growing clinical applications and success of adoptive cellular therapy of cancer has been due to the rapid evolution of immunology, gene therapy and stem cell biology and the translation of advances in these fields from the research laboratory to the clinic. The continued development of this field is dependent on the exchange of ideas across these diverse disciplines, the testing of new ideas in the research laboratory and in animal models, the development of new cellular therapies and GMP methods to produce these therapies, and the testing of new adoptive cell therapies in clinical trials. The Summit on Cell Therapy for Cancer will include a mix of perspectives, concepts and ideas related to adoptive cellular therapy that are not normally presented together at any single meeting. We hope that this novel assembly will generate new ideas and new collaborations and possibly increase the rate of advancement of this field.

## Competing interests

The authors declare that they have no competing interests.

## Authors' contributions

CJMM and JJO helped plan and write the manuscript. DFS helped plan the manuscript and drafted the manuscript. All authors have read and approved the final manuscript.
